# Cometabolic Degradation of Naproxen by *Planococcus* sp. Strain S5

**DOI:** 10.1007/s11270-015-2564-6

**Published:** 2015-08-14

**Authors:** Dorota Domaradzka, Urszula Guzik, Katarzyna Hupert-Kocurek, Danuta Wojcieszyńska

**Affiliations:** Department of Biochemistry, Faculty of Biology and Environmental Protection, University of Silesia in Katowice, Jagiellońska 28, 40-032 Katowice, Poland

**Keywords:** *Planococcus*, Cometabolism, Biodegradation, Naproxen, Dioxygenase, Monooxygenase

## Abstract

Naproxen is a non-steroidal anti-inflammatory drug frequently detected in the influent and effluent of sewage treatment plants. The Gram-positive strain *Planococcus* sp. S5 was able to remove approximately 30 % of naproxen after 35 days of incubation in monosubstrate culture. Under cometabolic conditions, with glucose or phenol as a growth substrate, the degradation efficiency of S5 increased. During 35 days of incubation, 75.14 ± 1.71 % and 86.27 ± 2.09 % of naproxen was degraded in the presence of glucose and phenol, respectively. The highest rate of naproxen degradation observed in the presence of phenol may be connected with the fact that phenol is known to induce enzymes responsible for aromatic ring cleavage. The activity of phenol monooxygenase, naphthalene monooxygenase, and hydroxyquinol 1,2-dioxygenase was indicated in *Planococcus* sp. S5 culture with glucose or phenol as a growth substrate. It is suggested that these enzymes may be engaged in naproxen degradation.

## Introduction

Increasing interest in non-steroidal anti-inflammatory drugs (NSAIDs) as pollutants of the aquatic environment has been observed during the last decade. After the publication of an article by Ternes in 1998, where the presence of 32 pharmaceuticals and their metabolites in an aquatic environment was described, a significant increase in the number of studies in which the authors indicated pharmaceuticals in soil samples, sediments, stream and river waters, wastewater treatment plants, and even drinking water has been carried out. NSAIDs have been detected in the environment in concentrations ranging from nanogram per liter to microgram per liter (Ternes 1998; Heberer 2002; Grenni et al. 2013; Grossberger et al. 2014). Many of them are slowly metabolized in the environment because of their persistence. They are excreted as demethylated and hydroxylated derivatives, acyl glucuronide conjugates, or parent drugs which accumulate in the environment (Vree et al. 1993; Christen et al. 2010). Non-steroidal anti-inflammatory drugs are the main inhibitors of cyclooxygenases. These enzymes characterize high homology regardless of organism ancestry. Due to this homology, NSAIDs may cause adverse effects in non-target organisms. Generally, environmental risk assessments are based on acute toxicity tests, which are carried out for higher concentrations of NSAIDs than those observed in the environment. However, there is little information about the chronic toxicity of environmentally relevant concentrations of these drugs. Accumulation of diclofenac in the liver, kidney, gills, and muscle of rainbow trout was reported by Schwaiger et al. (2004), Straub and Steward (2007), and Christen et al. (2010), while Melvin et al. (2014) suggested a minor influence of the exposure to low concentrations of NSAID mixtures on amphibian development. Due to the contamination of the environment by pharmaceuticals, it is necessary to find a way for their efficient degradation.

It is known that naproxen may be removed from the environment by physicochemical processes. However, during these processes, radical intermediates and/or secondary pollutants may be formed (Zhang et al. 2013). For example, Marotta et al. (2013) observed photolysis of naproxen to 1-(6-metoxy-2-naphthyl)ethanol and 2-acetyl-6-methoxynaphthalene. Moreover, physicochemical processes are very expensive and require harsh reaction conditions (Zhang et al. 2013). Bioremediation processes are a good alternative to above methods. However, effective degradation of naproxen in biological systems was not observed. Until now, only Wojcieszyńska et al. (2014) observed transformation of naproxen with about 28 % efficiency in monosubstrate culture. During removal of toxic and stable compounds, more effective are cometabolic systems. Cometabolism is defined as transformation of difficult to degrade and very stable xenobiotic compounds (cometabolite) simultaneously with degradation of easy to assimilate organic substrates (growth substrate) (Rieger et al. 2002; Greń et al. 2010). Introduction of growth substrate into the culture leads to cofactor synthesis, which are necessary for cometabolite degradation, and biomass production. Moreover, if the structure of growth substrate is similar to cometabolite, enzymes required for cometabolite transformation will be induced (Cornelissen and Sijm 1996). Schmidt et al. (1987) showed that degradation of resistant nitrophenols was more efficient in the presence of readily degraded organic substrates. It is known that naproxen exist in the environment together with either toxic or non-toxic substances which can provide sources of carbon, nitrogen, and energy. Therefore, the drugs can be more easily utilized by microorganisms colonizing polluted areas. Naproxen, as a polycyclic NSAID and naphthalene derivative, is more difficult to biodegrade by microorganisms like fungi and bacteria than monocyclic NSAIDs (e.g., paracetamol or ibuprofen). Quintana et al. (2005) observed the transformation of naproxen only in a cometabolic system. The utilization of this drug without an additional source of carbon did not occur. Moreover, the cometabolic degradation of naproxen in the presence of additional carbon sources was observed in our earlier work on the degradation of this drug by *Stenotrophomonas maltophilia* KB2 (Wojcieszyńska et al. 2014).

It was shown that non-steroidal anti-inflammatory drugs including naproxen were detected in the effluents of sewage treatment plants (Nakada et al. 2005; Brun et al. 2006; Brozinski et al. 2013). For example, in Canadian Sewage Treatment Plants effluents, naproxen was detected at concentration of 0.2–14 μg/L (Brun et al. 2006). Moreover, naproxen was detected in bream and roach bile caught downstream of a wastewater treatment plant (Brozinski et al. 2013).

Due to the increasing consumption and inefficient removal of naproxen in sewage treatment plants as well as accumulation of this drug and its metabolites in organisms, it is in urgent need of searching for microorganisms able to mineralize naproxen. The present work is a continuation of studies on the identification of bacterial strains able to efficiently utilize NSAIDs. This is the first study on the Gram-positive strain *Planococcus* sp. S5 engaged in the degradation of naproxen under cometabolic conditions. The identification of enzymes involved in naproxen degradation is also presented.

## Material and Methods

### Bacterial Strain and Chemicals

The *Planococcus* sp. strain S5 (GenBank AY028621) used in this study was isolated from activated sludge from the sewage treatment plant in Bytom Miechowice, Poland (Łabużek et al. 2003). It was cultivated in nutrient broth at 30 °C with agitation at 130 rpm for 24 h. After this time, 6 mg/L of naproxen was added to the culture. After 48 h, bacterial cells were harvested by centrifugation (5000×*g* at 4 °C for 15 min), washed with a sterile mineral salt medium, and used as an inoculum in experiments on the degradation of naproxen.

Naproxen was obtained from Sigma-Aldrich (USA). Acetonitrile and 1 % acetic acid were HPLC grade. Phenol was obtained from Merck (Germany), and glucose was purchased from POCH (Poland). All other chemicals used were of analytical grade.

### In Vivo Degradation Studies

Biological degradation of naproxen in monosubstrate and cometabolic systems was carried in 500-mL Erlenmeyer flasks containing 250 mL of a sterile mineral salt medium, which consisted of (g/L): Na_2_HPO_4_·12H_2_O 3.78, KH_2_PO_4_ 0.5, NH_4_Cl 5.0, MgSO_4_·7H_2_O 0.2, and 0.01 yeast extract. The inoculum of bacterial cells prepared as described above was added to the medium to an initial optical density of about 1.4 and 0.05 at *λ* = 600 nm for the monosubstrate and cometabolic systems, respectively. Additionally, two control cultures (250 mL) were prepared: an uninoculated control consisted of the mineral salt medium only, and a heat-killed control consisted of bacterial cells destroyed by autoclaving. The optical density of the heat-killed control was the same as for the examined cultures.

Naproxen was added to each flask to the final concentration of 6 mg/L. In the monosubstrate system, naproxen was the sole carbon and energy source, while for the cometabolic transformation of naproxen, 282.33 mg/L of phenol or 1 mg/L of glucose was added as growth substrates. When the complete degradation of growth substrate and OD_600_ < 0.8 was observed, the suitable growth substrate was added to the culture. All cultures were grown in triplicate and incubated with shaking at 130 rpm at 30 °C for 35 days.

### Determination of Substrate Concentration

Naproxen concentration was verified using HPLC (Merck HITACHI) equipped with a LiChromospher® RP-18 column (4 × 250 mm), liChroCART® 250-4 Nucleosil 5 C18, and a DAD detector. The mobile phase consisted of acetonitrile and 1 % acetic acid (50:50 *v*/*v*) at a flow rate of 1 mL/min. The detection wavelength was set at 260 nm (Wojcieszyńska et al. 2014). One-milliliter samples were taken every 7 days and centrifuged. Naproxen in supernatant was identified by comparison of HPLC retention time and UV-visible spectra with those of the external standards.

The concentration of phenol and glucose in supernatants was determined by the colorimetric method, with *p*-nitroaniline (Łurie and Rybnikova 1974) and 3,5-dinitrosalicylic acid, respectively (Miller 1959).

### Enzyme Assays

After 35 days of incubation, cells of *Planococcus* sp. strain S5 were harvested by centrifugation (4500×*g* for 15 min at 4 °C) and the obtained pellet was washed with 50 mM phosphate buffer, pH 7.0. Subsequently, the cells were disrupted by sonication (6 times for 15 s) and centrifuged at 9000×*g* for 30 min at 4 °C. The obtained crude extract was used for the enzyme assays. The activity of monooxygenase was determined spectrophotometrically by measuring NADH oxidation (*λ* = 340 nm; *ε* = 6220/M cm) (Divari et al. 2003). The naphthalene 1,2-dioxygenase, hydroxyquinol 1,2-dioxygenase, catechol 1,2-dioxygenase, and catechol 2,3-dioxygenase activity was measured spectrophotometrically by the formation of *cis*, *cis*-dihydrodiol (*λ* = 262 nm; *ε* = 8230/M cm), maleylacetate (*λ* = 243 nm; *ε* = 44,520/M cm), *cis*, *cis*-muconic acid (*λ* = 260 nm; *ε* = 16,800/M cm), and 2-hydroxymuconic semialdehyde (*λ* = 375 nm; *ε* = 3600/M cm), respectively (Cidaria et al. 1994; Wei et al. 2010; Wojcieszyńska et al. 2011). In order to determine gentisate dioxygenase, the formation of maleylpyruvate (*λ* = 330 nm; *ε* = 1080/M cm) was indicated (Feng et al. 1999). Protein concentration was measured by the method of Bradford (1976). One unit of enzyme activity was defined as the amount of enzyme required to generate 1 μmol of product per minute.

### Statistical Analysis

All experiments were performed in triplicate. The values of enzyme activities were analyzed by one-way ANOVA (*p* < 0.05) using STATISTICA 10.0 PL software package.

## Results and Discussion

### Biodegradation of Naproxen by *Planococcus* sp*.* S5

In recent years, biological methods have been increasingly used for the removal of various xenobiotics from the environment. Microorganisms possessing the efficient enzymatic systems are capable of degrading non-steroidal inflammatory drugs such as naproxen. To date, the transformation of this drug has mainly been observed in white-rot fungi (WRF).

Naproxen at a concentration of 10 mg/L and 55 μg/L was removed by *Trametes versicolor* after 6 and 5 h of incubation, respectively, while *Phanerochaete chrysosporium* was able to utilize 1 mg/L of the drug during 4 days of incubation (Marco-Urrea et al. 2010; Rodarte-Morales et al. 2011). Degradation of this pharmaceutical by activated sludge, biosolids, and mixed cultures of soil and river water has also been described (Quintana et al. 2005; Monteiro and Boxal 2009; Grenni et al. 2013). Lin et al. (2015) showed that *Pseudomonas* sp. CE21, a cefalexin-degrading bacterium isolated from the activated sludge, was also capable of eliminating sulfamethoxazole and naproxen to some extent.

**Fig. 1:**
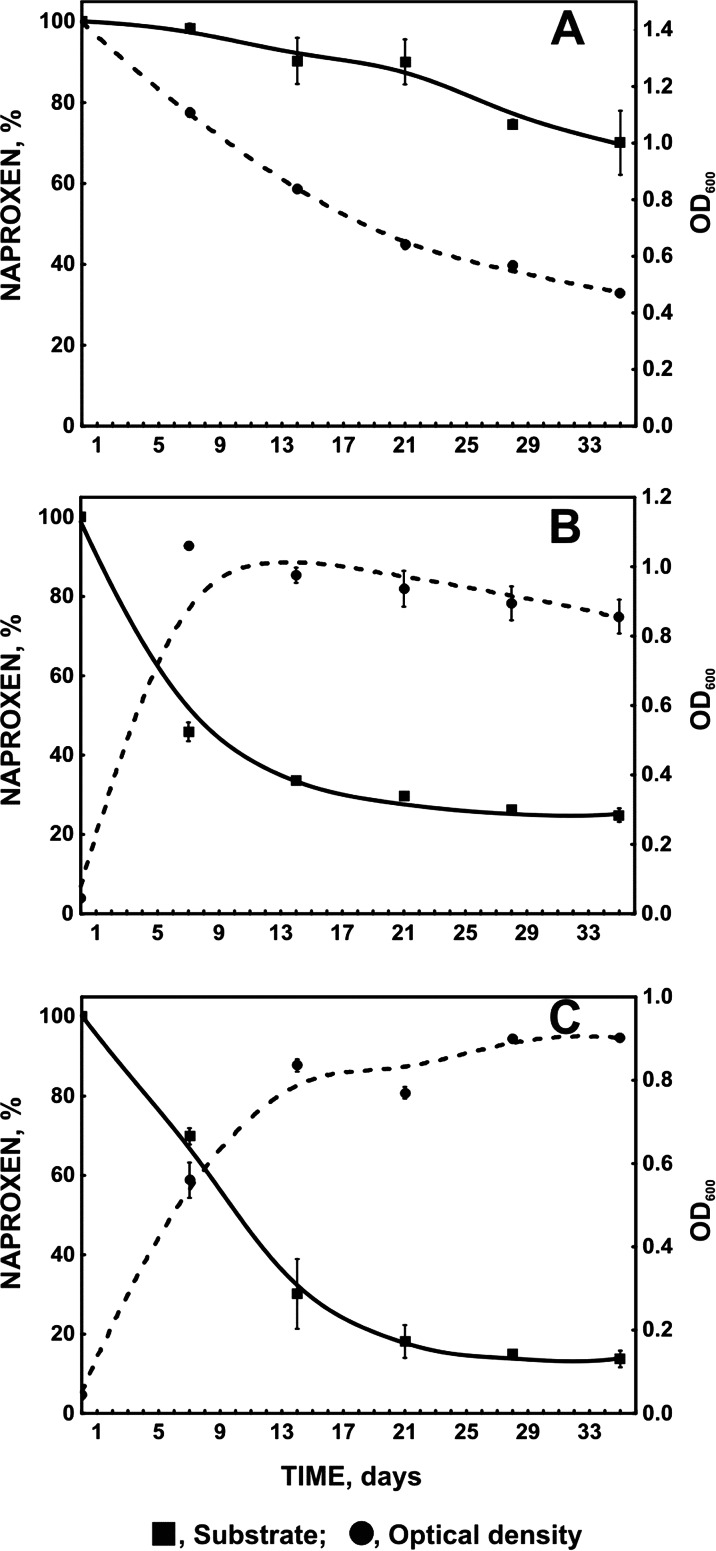
Degradation of 6 mg/L naproxen by *Planococcus* sp. strain S5 and changes of microbial biomass monitored as optical density at 600 nm (**a** without additional carbon source, **b** with 1 mg/L glucose as a simple carbon source, **c** with 282.33 mg/L phenol as a carbon source)

Gram-negative bacteria are more tolerant to xenobiotics than Gram-positive ones, mainly because of differences in the bacterial cell wall structure (Isken and Bont 1998; Greń et al. 2012). Although Gram-positive bacteria do not have additional permeability barriers, especially the outer membrane, some Gram-positive strains able to degrade aliphatic and aromatic hydrocarbons, antibiotics, and chlorophenols derivatives have been reported (Park et al. 2001; Rusnak et al. 2001; Lǎzǎroaie 2010; Shen et al. 2012). *Planococcus* sp. S5 is a Gram-positive strain known to degrade the aromatic structure of salicylate, benzoate, hydroxybenzoate, dihydroxybenzoate, and phenol (Łabużek et al. 2003; Hupert-Kocurek et al. 2012). Due to the degradation potential of this strain and the high activity of its catechol 2,3-dioxygenase strain, S5 was used for the degradation of naproxen (Hupert-Kocurek et al. 2012). In the first set of experiments, naproxen was used as a single substrate at a concentration of 6 mg/L. After 35 days of incubation, *Planococcus* sp. strain S5 removed approximately 29.93 ± 7.94 % of this substrate (Fig. 1a). However, the decrease in the optical density of the culture (from 1.4 to 0.5) indicates that naproxen was not a sufficient carbon and energy source for the strain. There were no changes in naproxen concentration in the controls. The results obtained for the uninoculated as well as the heat-killed control indicated that the pharmaceutical was eliminated from the culture by the biological way only. Low levels of naproxen degradation were also observed by Lin et al. (2015) and Wojcieszyńska et al. (2014). *Pseudomonas* sp. strain CE21 was able to degrade 78 % of 0.01 mg/L and 100 % of 0.1 mg/L of naproxen during 3 days (Lin et al. 2015). *S. maltophilia* strain KB2 degraded 28 % of 6 mg/L of naproxen during 35 days (Wojcieszyńska et al. 2014).

### Cometabolic Degradation of Naproxen by *Planococcus* sp. S5

Some of the microorganisms are able to degrade xenobiotics only under cometabolic conditions (Annweiller et al. 2001; Greń et al. 2010; Arulazhagan et al. 2014). The addition of a cosubstrate which is a readily available source of carbon and energy increases biomass production. The presence of the growth substrate may also induce enzymes of xenobiotic decomposition pathways and in this way increase the xenobiotic degradation rate (Schmidt et al. 1987; Quintana et al. 2005; Greń et al. 2012). For example, the addition of powdered milk as an external carbon source allowed the degradation of three acidic pharmaceuticals by activated sludge (Quintana et al. 2005). Under cometabolic conditions, about 30 % removal of bezafibrate, 49 % removal of naproxen, and 96 % removal of ibuprofen were observed after 26 days of the experiment, while in monosubstrate systems these drugs were not degraded (Quintana et al. 2005).

In the cometabolic cultures with glucose or phenol as an additional carbon source, the growth of the biomass of *Planococcus* sp. S5 was observed (from 0.05 to 0.8 or from 0.05 to 0.9 in the presence of glucose or phenol, respectively) (Fig. 1b, c). Moreover, under this condition, significant improvement of naproxen degradation efficiency was observed. 75.14 ± 1.71 % and 86.27 ± 2.09 % of naproxen was removed after 35 days of incubation in cultures with glucose or phenol as the carbon source, respectively (Fig. 1b, c). The calculated specific naproxen degradation rates for strain S5 grown in the presence of glucose or phenol as growth substrates were 19 and 11 μg/h, respectively, whereas the specific naproxen degradation rate in monosubstrate culture was 0.5 μg/h. These results indicate that easily assimilable carbon source significantly accelerates initial degradation velocity. Glucose used as growth substrate stimulated the degradation of non-steroidal anti-inflammatory drugs by *P. chrysosporium.* Eighty-three percent of naproxen was transformed after 23 h (Rodarte-Morales et al. 2012). The other cosubstrate we used for the degradation study of naproxen was phenol. In the presence of this aromatic compound, the largest decrease in naproxen concentration was determined (Fig. 1c). Conversely, in our studies on naproxen degradation by *S. maltophilia*, degradation of this drug was most effective in the presence of glucose as a growth substrate (Wojcieszyńska et al. 2014). The highest percentage of naproxen degradation observed in the present study could be explained by the fact that phenol is known to induce enzymes responsible for aromatic ring cleavage (Krastanow et al. 2013). In the natural environment, xenobiotic compounds are present together with natural growth substrates. Therefore, cometabolic systems better reflect the degradation processes that take place in nature.

### Activation of Enzymes During Cometabolic Degradation of Naproxen

In the degradation of naproxen by white rot fungi laccases, and peroxidases like lignin peroxidases, manganese-dependent peroxidases and versatile peroxidases are engaged (Pointing 2001; Lloret et al. 2010; Marco-Urrea et al. 2010; Rodriguez-Rodriguez et al. 2010a, b; Tran et al. 2010). The formation of naproxen metabolite-1-(6-metoxynaphthalen-2-yl) ethanone as a result of laccase activity was observed by Marco-Urrea et al. (2010), whereas manganese peroxidase activity during this drug degradation was shown in *Bjerkandera* and *P. chrysosporium* (Rodarte-Morales et al. 2011). An alternative mechanism of non-steroidal anti-inflammatory drug degradation involves intracellular cytochrome P-450. It plays a key role in 6-desmethylnaproxen formation in cultures of *T. versicolor* and *P. chrysosporium*. Cytochrome P-450 and detoxification enzymes analogous to mammal enzymes of phase II were engaged in the formation of 6-desmethylnaproxen and desmethylnaproxen-6-*o*-sulfate during the transformation of naproxen by *Cunninghamella* sp. (Zhong et al. 2003; Marco-Urrea et al. 2010; Rodarte-Morales et al. 2011; Rodarte-Morales et al. 2012). Conversely, there is little information about enzymes engaged in the degradation of naproxen by bacteria. In our earlier work, we indicated the contribution of naphthalene dioxygenase and phenol monooxygenase as hydroxylating enzymes, and hydroxyquinol 1,2-dioxygenase and gentisate 1,2-dioxygenase as aromatic ring cleaving enzymes in the degradation of naproxen by *S. maltophilia* strain KB2 (Wojcieszyńska et al. 2014).

Naproxen is one of the naphthalene derivatives. Therefore, during its degradation, the induction of enzymes involved in the degradation of polycyclic aromatic compounds was expected. Because of low biomass concentration in the culture with naproxen as the sole carbon and energy source, enzymes were isolated from cultures supplemented with glucose or phenol as cosubstrates. The activity of phenol monooxygenase, naphthalene dioxygenase, and hydroxyquinol 1,2-dioxygenase observed in *Planococcus* sp. S5 cultures either with glucose or phenol (Tab. 1) suggested the participation of these enzymes in naproxen degradation. These results may also indicate that hydroxylation is the first step in naproxen degradation. Generally, naphthalene dioxygenase hydroxylates C1 and C2 of naphthalene (Jeffrey et al. 1975). However, due to the propionic chain at C2 of naproxen, naphthalene dioxygenase in *Planococcus* sp. S5 may catalyze hydroxylation of this compound at C7 and C8 positions. The activity of phenol monooxygenase indicates the possibility of a third hydroxylation of the aromatic ring. The intradiol cleavage of the ring between C7 and C8 catalyzed by hydroxyquinol 1,2-dioxygenase is suggested to be the second step of naproxen degradation by Gram-positive strain S5. These two steps (hydroxylation and aromatic ring fission) are typical for xenobiotic degradation strategy in bacteria (Latus et al. 1995; Perpetuo et al. 2009; Guzik et al. 2013).

**Table 1:** Specific activity of enzymes engaged in naproxen degradation under cometabolic conditions

Enzyme	Specific enzyme activity (U/mg protein)	
	Naproxen + glucose	Naproxen + phenol
Phenol monooxygenase	28.32 ± 7.5	34.70 ± 5.8
Naphthalene dioxygenase	1.36 ± 1.0*	4.74 ± 1.0*
Hydroxyquinol 1,2-dioxygenase	190.80 ± 79.4	250.67 ± 18.8
Catechol 1,2-dioxygenase	0.0 ± 0.0	0.0 ± 0.0
Catechol 2,3-dioxygenase	0.0 ± 0.0*	1070.62 ± 5.7*
Gentisate 1,2-dioxygenase	517.98 ± 18.3*	0.0 ± 0.0*

*Values of enzyme activities which differ significantly (*p* < 0.05) in dependence on the growth substrate

The key intermediate in naphthalene metabolism is salicylate, which can be cleaved by the *ortho* or *meta* pathway enzymes (Seo et al. 2009). Because in the presence of sodium salicylate, in *Planococcus* sp. S5 activity of catechol 2,3-dioxygenase as well as catechol 1,2-dioxygenase was observed (Łabużek et al. 2003), these enzymes were expected to be involved in naproxen degradation. Isolation and determination of catechol dioxygenase activity revealed the activity of catechol 2,3- dioxygenase in the culture with phenol as a growth substrate (Tab. 1), while in the culture with naproxen and glucose as a cosubstrate no activity of this enzyme was observed (Tab. 1). There was no activity of catechol 1,2-dioxygenase in cometabolic systems with phenol or glucose as growth substrates (Tab. 1). Catechol 2,3- dioxygenase of *Planococcus* sp. S5 is known to be induced in the presence of phenol (Hupert-Kocurek et al. 2012). Therefore, the activity of this enzyme only in the system with naproxen and phenol indicates its engagement in the cleavage of dihydroxylated derivative of phenol rather than in naproxen degradation. In the presence of glucose as a cosubstrate, the activity of gentisate 1,2-dioxygenase was observed (Tab. 1). It is known that salicylate can undergo transformation to gentisate, which is then cleaved by gentisate 1,2-dioxygenase to maleylpyruvic acid (Seo et al. 2009). The activity of gentisate 1,2-dioxygenase in the *Planococcus* sp. S5 culture with naproxen and glucose indicates that naproxen degradation is a more complicated process than was thought.

## Conclusion

Apart from white-rot fungi, bacteria are potent microorganisms with which to degrade and remove non-steroidal anti-inflammatory drugs from the environment. Under cometabolic conditions, the Gram-positive bacterium *Planococcus* sp. S5 degraded naproxen with degradation rates 19 and 11 μg/h in the presence of glucose and phenol, respectively. In the presence of easily assimilable source of carbon, faster degradation of naproxen was observed. For that reason, selection of proper conditions (e.g., kind and concentration of the carbon source) may reduce the treatment time. The activity of hydroxylating and aromatic-ring-cleaving enzymes confirms their key role in pathways of microbial degradation of naproxen. Obtained results suggest the possible application of cometabolic systems for the treatment of naproxen burdened wastewater. It should be noticed that the occurrence of naproxen in environmental waters is in the range of nanogram per liter to microgram per liter. Therefore, further naproxen biodegradation tests by *Planococcus* sp. S5 at the level of microgram per liter of naproxen would be necessary.
